# Validation and Improvement of a Bicycle Crank Arm Based in Numerical Simulation and Uncertainty Quantification

**DOI:** 10.3390/s20071814

**Published:** 2020-03-25

**Authors:** R. Gutiérrez-Moizant, M. Ramírez-Berasategui, José A. Calvo, Carolina Álvarez-Caldas

**Affiliations:** Mechanical Engineering Department, Universidad Carlos III de Madrid, Leganés, 28911 Madrid, Spain; mrami@ing.uc3m.es (M.R.-B.); jacalvo@ing.uc3m.es (J.A.C.); calvarez@ing.uc3m.es (C.Á.-C.)

**Keywords:** crank arm, strain gauges, uncertainty propagation, metamodel, statistical validation, fatigue test

## Abstract

In this study, a finite element model of a bicycle crank arm are compared to experimental results. The structural integrity of the crank arm was analyzed in a universal dynamic test bench. The instrumentation used has allowed us to know the fatigue behavior of the component tested. For this, the prototype was instrumented with three rectangular strain gauge rosettes bonded in areas where failure was expected. With the measurements made by strain gauges and the forces registers from the load cell used, it has been possible to determine the state of the stresses for different loads and boundary conditions, which has subsequently been compared with a finite element model. The simulations show a good agreement with the experimental results, when the potential sources of uncertainties are considered in the validation process. This analysis allowed us to improve the original design, reducing its weight by 15%. The study allows us to identify the manufacturing process that requires the best metrological control to avoid premature crank failure. Finally, the numerical fatigue analysis carried out allows us to conclude that the new crank arm can satisfy the structural performance demanded by the international bicycle standard. Additionally, it can be suggested to the standard to include the verification that no permanent deformations have occurred in the crank arm during the fatigue test. It has been observed that, in some cases this bicycle component fulfils the minimum safety requirements, but presents areas with plastic strains, which if not taken into account can increase the risk of injury for the cyclist due to unexpected failure of the component.

## 1. Introduction

The cycling industry has experienced a breakthrough in recent years. The demand for increasingly demanding performance—both in the professional and amateur sector—and the inclusion of the bicycle in the city as a clean, silent, inexpensive and healthy personal transport, are responsible for the technological growth of the industry [1]. In professional cycling, it is desired to achieve minimum weight and high rigidity of the structure to improve the performance of the elite athletes [2]. Materials such as aluminum alloy, titanium and carbon fiber are used for manufacturing several of the structural elements of the bicycle [2,3,4]. Moreover, every development performed on the bicycle has to take into account the security of the cyclist, because any unexpected failure might cause serious injuries to the rider [5,6]. To ensure the structural durability requirements of bicycle components, the European Committee for Standardization (CEN) developed the standard EN 14781: 2006. This document provides the safety requirements and test conditions applicable to the design and manufacture of race bicycles. [7]. The adoption and development of the recommendations given by the standard ensures that all the bicycle components are tested with the same method and guarantee the higher quality and safety of the bicycle as a whole [6].

Currently, the study of cycling can be divided into two research groups: One that seeks to optimize the structural components of the bicycle and another dedicated to the study of the biomechanical system formed by the cyclist and the bicycle.

The structural optimization of the bicycle include the study of the frame [3,4,8], the numerical and experimental analysis of the crank [2,5,9,10] and the measure of the operational forces acting in different components of the bicycle [6,11,12].

One of the most important components in the bicycle is the crank arm. It transmits the force that the cyclist applies to the pedal to the crank set. The strength of the material used in its manufacturing should resists the application of combined flexion and torsion loads without failure [2,10]. Its damage is produced by the progressive stiffness deterioration due to the operational loadings effect, which produces an unexpected failure [7]. According to the European Committee for Standardization [7], the crank arm can be considered appropriate for a professional use if it is able to withstand 100,000 load cycles without failure. However, the standard does not require to check the existence of plastic strains on the potential failure zone during the test. Aluminum 7000 alloys (rolled or cold-finished bar) at room temperature are the preferred material for the manufacturing of cranks, because of their low cost, high machinability and excellent performance with cyclic loading [5]. 

The use of computer simulation models in all engineering fields are reducing the design cycle time and cost, without decreasing the reliability of the final products. However, it is necessary to validate the computational model with experiments [13]. The validation procedure allows the quantifying of the confidence level of the model and to test the quality of the prediction made [14]. For statistical model validation, the uncertainty quantification is mandatory to quantify the accuracy of the mathematical model solution with respect to the real case under study. On the other hand, the model comparison is the evaluation of the behavior of the computational model with respect to the real case under study with other boundary conditions [13]. 

To reduce the number of simulations required to quantify the uncertainty of the complex computational model, a subrogate model can be used [15]. This subrogate model known as the metamodel represents the behavior of a zone of interest in the computational model and allows to identify the principal variables of influence of the system [16]. The metamodels are used to understanding the physical system analyzed, predicting its response. Moreover, it allows optimizing of the design, simplifying the validation procedure [15].

In modern engineering, it is necessary to identify all possible sources of uncertainty, since with this there is a better control of both the experimental test and the theoretical model to be validated. In the different studies carried out to date of bicycle components, the possible sources of uncertainty in the validation of the results had not been considered. Additionally, the effect of the uncertainty quantification in the optimization or improvement of the component constitutes a novelty.

In this research work, an aluminum 7000-T6 alloy crank arm has been experimentally tested under cycling loads in a universal testing machine. The crank has been instrumented with three rectangular strain gauge rosettes bonded in the potential failure areas [10]. The use of this type of sensor is justified since the location of the potential failure zones are known. In addition, it does not introduce additional stresses in the studied area and although it is not an ideal strain sensor, its sources of error are known and therefore can be calculated and extracted algebraically from the measured strain readings [17]. Therefore, accurate measurements can be obtained accompanied by their corresponding expanded uncertainty for the confidence level required. Additionally, a numerical model of the specimen has been developed with the finite element software Abaqus. Experimental results allow validation of the finite element model, where the potential sources of uncertainty are taken into account. To do that, it has been necessary to obtain a metamodel for the same areas analyzed in the real model. This allows identification of the sources of uncertainty that affect most of the behavior of the crank arm. Once the model has been validated, the next step has been to analyze its structural behavior using the direct cyclic approach of Abaqus. According to the simulation results, it is possible to reduce the weight of the crank arm by 15% while still satisfying the safety requirement given by the European Committee for Standardization [7]. Moreover, it could be recommended to the European Committee for Standardization to require the verification that there is not permanent deformation at the end of the crank fatigue test. According to the simulation results, a crank arm can fulfil the minimum safety requirements, but present zones with plastic strain. This produces a residual stress that can increase the probability of premature crank arm failure and therefore the risk of injury for the rider.

## 2. Materials and Methods

### 2.1. Crank Arm

The analyzed bicycle crank has been manufactured from a bar of aluminum alloys 7000-T6. This is an aluminum and zinc alloy with excellent mechanical behavior for this type of scenario, subjected to fatigue conditions. Its properties are summarized in Table 1. 

Since the stress distribution in the bicycle crank arm is unknown, it is decided to instrument it with three rectangular stacked grid 3-element strain gauge rosettes, model FRA-2-23-1L of 2 mm grid. This strain gauge configuration is appropriate when the directions of the principal strains are unknown [17]. Additionally, the stacked version is recommended when the space available for gauge installation is small and possible steep strain gradient can be expected in the surface of the test part. For a given active gauge length, all the strain gauges of the stacked rosette have their geometric centers on the same test part point, so they experiment the same strain field [17].

**Figure 1:**
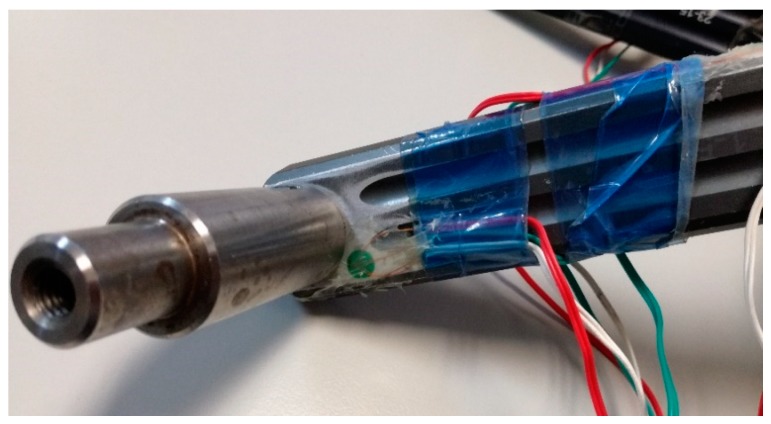
Position of the strain gauge rosette #1.

**Figure 2:**
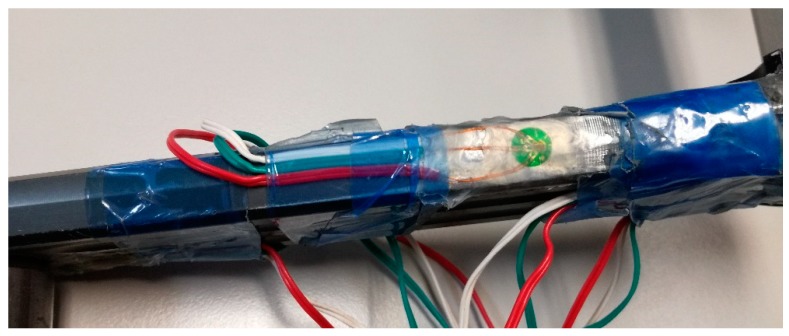
Position of the strain gauge rosette #2.

**Figure 3:**
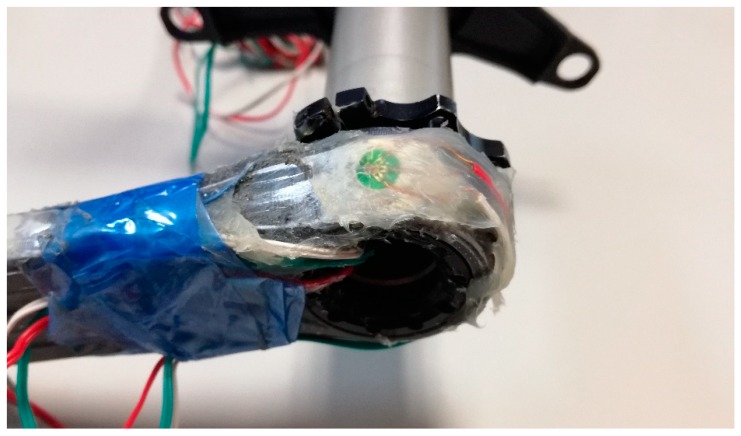
Position of the strain gauge rosette #3.

The rosettes have been fixed in those critical areas where the failure usually occurs, according to the observations of other researchers [2,10]. Figure 1, Figure 2 and Figure 3 show the distribution of the strain gauge rosettes on the tested crank arm.

**Figure 4:**
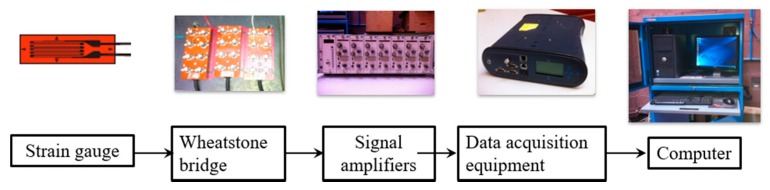
Representation of the measurement chain used for each strain gauge.

For all the strain gauges, an external Wheatstone quarter bridge of 120 Ω with a two lead-wire configuration has been settled and connected to several signal amplifiers (model NEC DC Strain Amplifier AS2101 manufactured by NEC SAN-EI Instruments, Ltd., Tokyo, Japan). The length of the lead-wires used in the strain gauges was short (50 cm), so it is concluded that the unbalanced condition in the bridge circuit can be neglected. However, the error produced by the lead-wire length has been mathematically subtracted from all the measured strain readings. The data acquisition system amplifies the signal from the strain gauges, while eliminating the signal noise. For that, the equipment used a low-pass filter of 10 Hz to all received signals before sending them to the digital signal converter. A calibration voltage of 10 V for all NEC SAN-EI channels was specified. This voltage value corresponds to a calibration strain set at 2000 µε. Afterwards, the measured strain values have been stored in an IMC CRONOS data acquisition equipment connected to a computer with a measurement tolerance of ±0.5 µε. Figure 4 shows the representation of the measurement system used for each strain gauge.

### 2.2. Crank Test Procedure 

**Figure 5:**
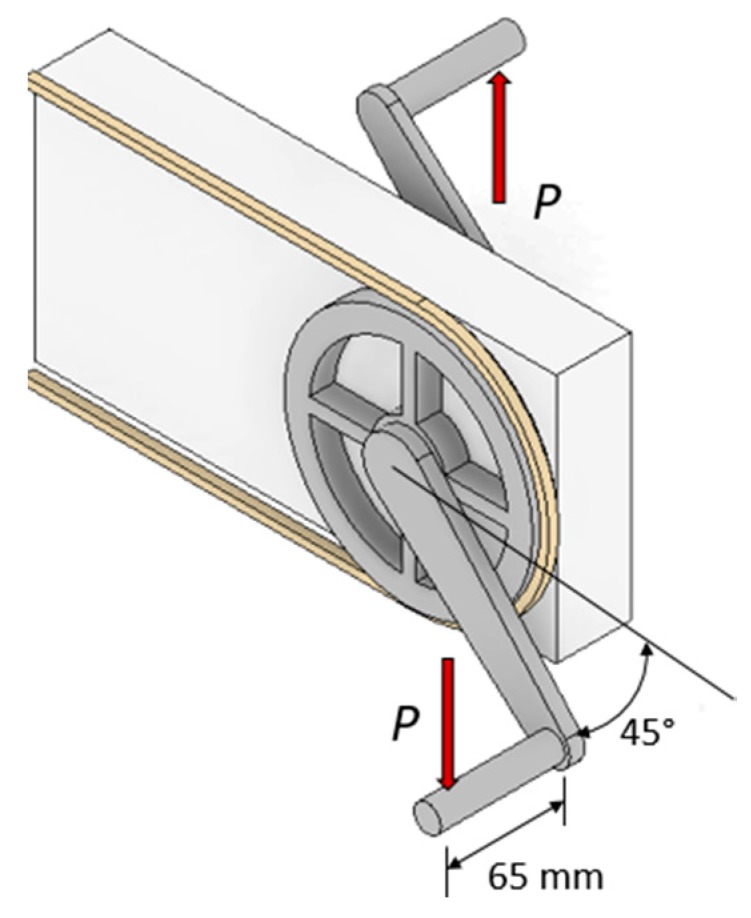
Bicycle crank test conditions according to EN 14781:2006 [7].

According to the standard [7], this bicycle component has to be subjected to a cycling load test in order to verify that it fulfils the minimum safety requirements. To carry on the test, the entire crank set, including the chain ring and the bottom bracket, has to be fixed to a device that represents the bicycle crank assembly. The crank arm should form 45 degrees with the horizontal plane, and the assembly has to be blocked with the chain in order to restrict the turn movement. The load has to be applied in a point placed on the pedal axle at 65 mm from the crank. For professional cycling cranks, the load varies from 0 to 1800 N with a maximum frequency of 25 Hz. The crank arm can be considered suitable if it is able to withstand 100,000 load cycles without failure. Figure 5 shows the schematic representation of the bicycle crank test according to the standard.

**Figure 6:**
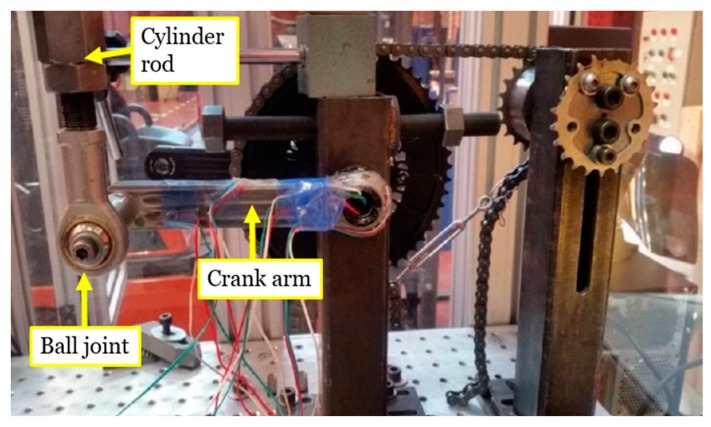
Bicycle crank test and its equipment.

Calvo et al. [10] analyzsed the influence of the variation of the test conditions given by the standard [7]. They observed that the angle between the crank and the horizontal plane has a great impact in the magnitude of the stress distribution in the crank. According to this research, the maximum stress was recorded between 0° and 30° around the zone of the strain gauge rosette #2 (Figure 2). This position range is the most critical in terms of fatigue life. Taking into account the suggestion given by Calvo [10], in the present research the crank arm has been tested at 0° in order to validate the developed computational model. Figure 6 shows the bicycle crank test and the used device.

**Figure 7:**
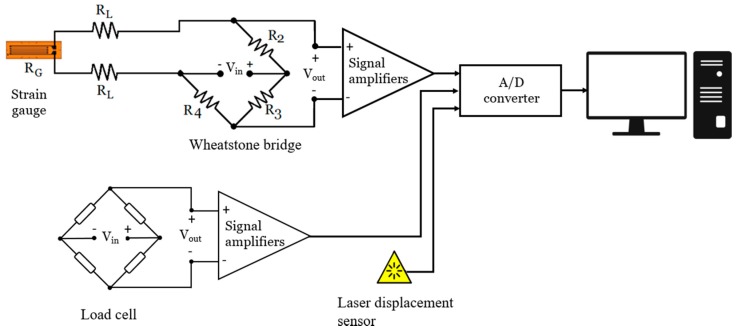
Schematic representation of the measurement system used in the test.

The crank arm was tested in an universal testing machine class II [19]. The load has been applied through the hydraulic actuator of the machine. For the load control, a load cell of 2500 N was used. The connection between the rod of the hydraulic cylinder and the axis of the pedal of the crank arm has been made with a ball joint (Figure 6), which guarantees the verticality of the axis of the cylinder rod and avoids transmitting reaction forces that could affect the normal operation of the testing machine. The employed data acquisition system is able to record at the same time all the strain measured by the strain gauges, the applied load and the displacement of the cylinder rod. The data recorded frequency was set at 1000 Hz. Figure 7 shows the schematic representation of the measurement system used in the test of the crank arm.

The structural analysis of the crank arm has been developed in two steps (statistical validation and single comparison). In the first step, ten tests were performed to validate the computational model. In these tests, the cycling loading varies from 0 to 918 N. Von Misses stresses were calculated from the measures registered by the strain gauges with their respective combined uncertainty. Finally, a fatigue test was carried out to confirm that the physical model meets the minimum safety requirements according to the standard [7].

### 2.3. Experimental Stress Determination

Once all the tests have been carried out, the errors of the strain measurement technique used have been quantified. The main error sources are shown in Table 2. 

When the errors produced by all the sources have been calculated, they can be subtracted from the final strains (1). Then, the corrected strains of each rosette have been transformed into principal strains (2).

(1) $$\varepsilon_{i}={\hat{\varepsilon }}_{i}-E_{W}-E_{T}-E_{TS},$$

(2) $$\varepsilon_{P,Q}=\frac{1}{2}\left\{\varepsilon_{1}+\varepsilon_{2}\pm \sqrt{2\left[{\left(\varepsilon_{1}-\varepsilon_{3}\right)}^{2}+{\left(\varepsilon_{2}-\varepsilon_{3}\right)}^{2}\right]}\right\},$$

where *ε*_1_, *ε*_2_ and *ε*_3_ are the corrected strain readings from gauge 1, 2 and 3 of the strain gauge rosettes, respectively.

The biaxial form of the Hooke’s law has been used for the calculation of the principal stresses (3) and (4), assuming that the material is homogenous and isotropic.

(3) $$\sigma_{P}=\frac{E}{1-\nu^{2}}\left(\varepsilon_{P}+\nu \varepsilon_{Q}\right),$$

(4) $$\sigma_{Q}=\frac{E}{1-\nu^{2}}\left(\varepsilon_{Q}+\nu \varepsilon_{P}\right),$$

where *E* is the Young Modulus and *ν* is the Poisson ratio of the crank arm (Table 1).

Finally, the equivalent Von Mises stress for each strain rosette has been obtained as shown in Equation (5).

(5) $$\sigma_{VM}=\sqrt{\sigma_{P}^{2}+\sigma_{Q}^{2}-\left(\sigma_{P}\cdot \sigma_{Q}\right)}.$$

#### 2.3.1. Procedure to Quantify the Experimental Stress Uncertainty

As it has been explained in the previous section, the final Von Mises stress is not directly measured; instead, it is calculated from other magnitudes, as strains experimented by the strain gauges, Young modulus and Poisson ratio of the material. Consequently, its uncertainty will be produced by a combination of different sources of error [20]. 

For the uncertainty quantification of the measurand, in this case the Von Mises stress, the law of propagation of uncertainty [21] can be used as well as alternative methods that use Monte Carlo simulations [22]. However, in both cases, it is necessary to know the probability distribution function of every considered error source. 

In this research the probable values of the Von Mises stress have been calculated using the Monte Carlo methods (MMC), following the supplements of the Guide to the expression of Uncertainty in Measurement (GUM) [22]. This method consists in generating a population of random data for each input variable, according to its probability density function, and then the output is calculated to estimate the mean, the standard deviation and its upper and lower limits for a confidence levels of 95%. In this case, for the Von Mises stress, 10^6^ reiterations of the Monte Carlo method have been simulated. A coverage interval of 95% has been considered [22]. The uncertainties values and the probability distribution functions (PDF) of each considered variable are shown in Table 3. Several uncertainties have been calculated by means of type B evaluation, i.e., assuming a symmetric rectangular distribution as recommended in the GUM [21,22]. The uncertainty of the load *P* corresponds to the value of a machine class II [19]. Moreover, conservative uncertainties of the elastic properties of the aluminum have been considered: 1% for the Young modulus *E* and 2% for the Poisson ratio *v*, with a confidence level of 95%.

**Figure 8:**
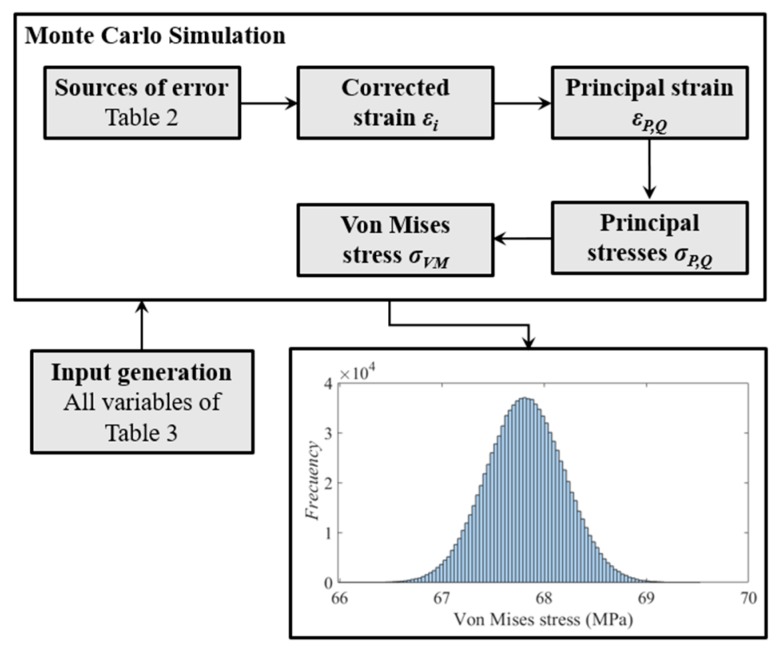
Uncertainty quantification of the Von Mises stress with the Monte Carlo method (MMC).

Figure 8 shows the schematics representation of the procedure followed for the calculus of the probable values of the Von Mises stress with the Monte Carlo Method.

The estimation of the magnitude of the Von Mises stress from the MMC ${\bar{\sigma }}_{VM}$ and its associated typical uncertainty for each strain gauge rosette $u\left({\bar{\sigma }}_{VM}\right)$ will be [22]:

(6) $${\bar{\sigma }}_{VM}=\frac{1}{M}\sum_{r=1}^{M}\sigma_{VM,r},$$

(7) $$u^{2}\left({\bar{\sigma }}_{VM}\right)=\frac{1}{M-1}\sum_{r=1}^{M}\left(\sigma_{VM,r}-{\bar{\sigma }}_{VM}\right),$$

where, *M* and *σ_VM,r_* are the number of MMC trials and the *r*th value of the Von Mises stress, respectively.

The standard uncertainty (9) associated to the final experimental Von Mises stress (8) considers the contribution due to the combined uncertainty of the strain gauge rosette (7) and the experimental standard deviation of the *n* = 10 repetition of the *h* = 10 loading cycles.

(8) $$\sigma_{VM\_EXP}=\frac{1}{n\cdot h}\sum_{i=1}^{n}\sum_{j=1}^{h}{\bar{\sigma }}_{VM(i,j)},$$

(9) $$u\left(\sigma_{VM\_EXP}\right)=\sqrt{\frac{1}{\left(n\cdot h\right)-1}\sum_{i=1}^{n}\sum_{j=1}^{h}{\left(\sigma_{VM\_EXP}-{\bar{\sigma }}_{VM(i,j)}\right)}^{2}},$$

## 3. Finite Element Model of the Crank Arm

**Figure 9:**
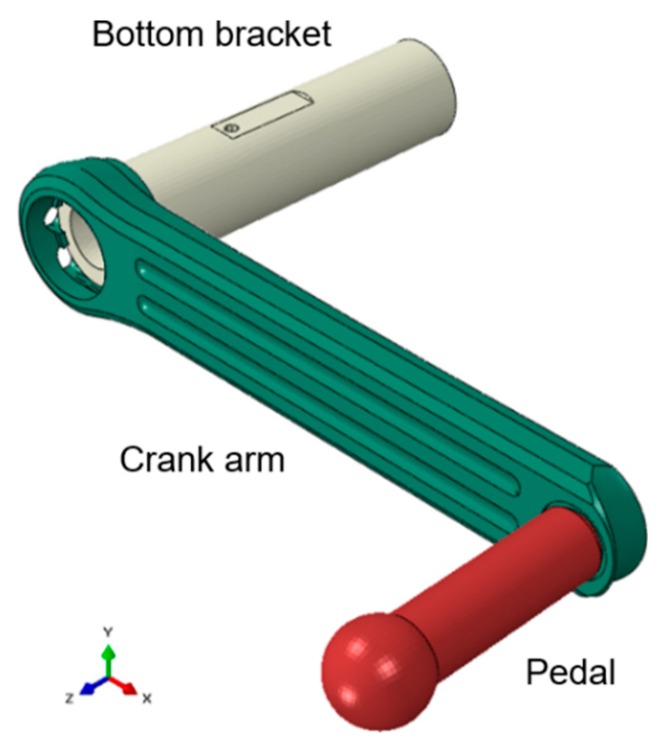
Finite element model of the crank arm assembly.

The numerical model analyzed in this paper has been performed in Abaqus. As the objective is that the boundary conditions are as similar as possible to the real test, it has been decided to include the axle of the bottom bracket and the axle of the pedal in the numerical analysis. The assembly of the components mentioned before is shown in Figure 9. The material properties of the crank arm and the axle of the bottom bracket correspond to the values of the aluminum alloy shown in Table 1. The axle of the pedal has been modelled as steel with a Young’s modulus of 210 GPa and a Poisson ratio of 0.33. As in the real assembly there is no relative movement between the parts, a tie type of contact has been defined between the parts in the numerical model. In order to recreate the experimental conditions, the extreme side of the bottom bracket axis that is not in contact with the crank arm, has been completely fixed. However, the rotation of this part around its axial axis of symmetry. Additionally, two symmetry surface partition have been defined along the axis of this component, these represent the area in contact with the rolling bearing. In this zone, only the turn movement around the axis is allowed.

All the components of the numerical model have been designed as similar as possible to the physical components. The axis of the pedal is the component that transfers the efforts to the rest of the set. As can be seen in Figure 9, it has been decided not to include the connection between the rod of the hydraulic cylinder and the axis of the pedal, i.e., the ball joint; because this only guarantees the normal operation of the testing machine but does not have significant effect on the crank arm behavior. The vertical load has been applied directly on the spherical surface of the pedal at the same distance as that required in the experimental test.

**Figure 10:**
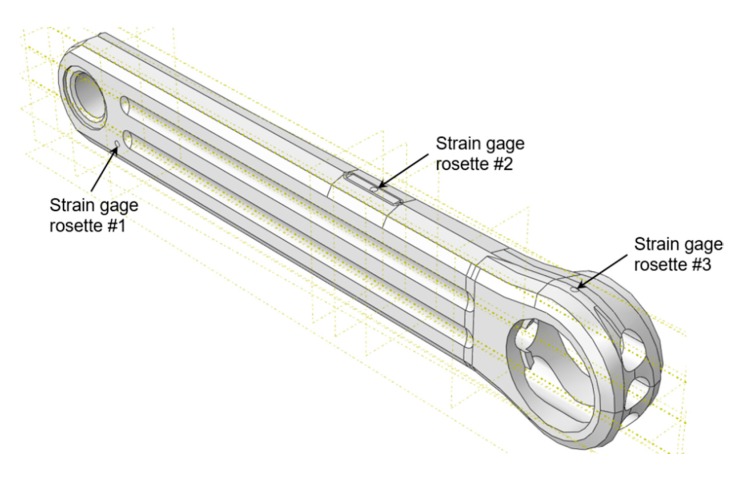
Finite element of the crank arm.

Extra partition zones were created in that areas occupied by the strain gauge rosettes (Figure 10), in order to reproduce their location in the real device. 

The model has been meshed with 123450 10-node quadratic tetrahedrons, because they adapt better to the complex analyzed geometry. The final size of the elements that guarantees the convergence of the simulation has been determined considering the computational time and the sensitivity of the mesh in the simulations results, (the variation of the results was less than 0.2% between the last consecutive simulations). The size of the elements is 1.5 mm, except in the partitions corresponding to the strain gauges rosettes (Figure 10), where a size of 0.1 mm has been selected. 

### 3.1. Uncertainty Quantification Procedure for the Finite Element Model

This part of the study will allow us to know the impact that the input parameters and the test conditions have on the structural behavior of the analyzed component. To analyze if the finite element model can adequately represent the real behavior of the crank arm it is necessary to compare the simulation results with the experimental test. The validation models allow to verify if the numerical solutions obtained with the model have enough credibility [23]. 

The input variables used in a finite element model always present some uncertainty, due to their stochastic nature. The uncertainty quantification related to numerical simulations does not yet have a standardized procedure, due to the computational efforts required [22]. 

To reduce the number of simulations necessary to assess the uncertainty of a finite element model, it is commonly accepted to use metamodels constructed from the results of the several simulations generated with the variation of the input parameters [14,15,16,24]. These surrogate models provide a computationally efficient analysis. It is assumed that they represent the solution of the finite element model for the areas under study. Moreover, the uncertainties of the variables used in its construction can be propagated through random simulations with the Monte Carlo method. Then, the mean magnitude, the standard deviation and coverage limits are calculated, generally for a confidence level of 95% [14].

The uncertainty quantification developed in this research work has been divided in two sections:

- The uncertainty due to the material properties (Young modulus, Poisson ratio) and the applied load.
- The uncertainty due to the most critical geometrical design tolerances commonly present in the manufacturing of the crank arm.

Three metamodels were constructed for each strain gauge rosette zone using design of experiments (DOE). The DOE is an experimental technique to analyze the influence of the inputs of a system in its final result. To do this, all the possible combinations of input factors have been considered, based on a discrete representation of their possible values. The approach allows to evaluate both the effect of each input factor, as well as the effects of interactions between them on the response of the system [25].

In all the simulations, the load applied takes into account the experimental deviation and the uncertainty of the universal testing machine. 

#### 3.1.1. Uncertainty Due to the Material Properties

In this section of the uncertainty quantification, a metamodel approach was constructed using a Central Composite Design (CCD). The levels of the CCD are shown in Table 4. The CCD is probably the most popular experiment design method because it allows to evaluate the simulation response in all directions of the parameter space [25]. The approximation relation between the inputs (Young modulus, Poisson ratio and applied load) and the Von Misses stress has been developed for the combination of the values of the expanded uncertainty of the inputs, considering a confidence level of 95%.

**Figure 11:**
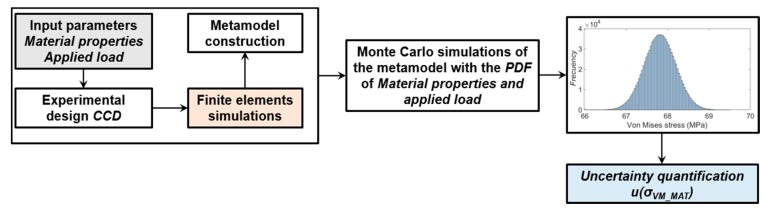
Uncertainty quantification procedure of the computational stress due to materials properties and load.

Finally, the uncertainty of each contribution shown in Table 3, has been propagated using the metamodel expression obtained from the CCD analysis. Statistical magnitudes as mean, uncertainty and confidence level of 95%, where calculated with a crude Monte Carlo simulation of 10^6^ random evaluations of the metamodel expression, according to GUM [22]. Figure 11 represents this procedure for the computational Von Mises stress due to the material properties and applied load.

The mean and associated typical uncertainty of the Von Mises stress can therefore be expressed by [22]:

(10) $$\sigma_{VM\_MAT}=\frac{1}{M}\sum_{r=1}^{M}\sigma_{VM\_MAT,r},$$

(11) $$u\left(\sigma_{VM\_MAT}\right)=\sqrt{\frac{1}{M-1}\sum_{r=1}^{M}\left(\sigma_{VM\_MAT,r}-\sigma_{VM\_MAT}\right)},$$

where, *M* and *σ_VM_MAT,r_* are the number of MMC trials and the *r*th probable value of the numerical Von Mises stress calculated, respectively.

#### 3.1.2. Uncertainty Due to the Geometrics Tolerance Properties

The uncertainty due to the dimensional tolerances of the real model can come from a large number of possible sources that depend on the complexity of the piece. In this section, it has been decided to quantify only those geometric tolerances that can be crucial in the manufacturing of the crank arm. In professional cycling, the aim is to increase the rigidity of each component of the bicycle, minimizing as much as possible their weight. The analyzed crank arm model has three blind central holes along almost its entire length. This machining process is considered to be the most critical phase of manufacturing, due to the variations of the nominal value of the drill’s diameters, as well as their symmetry and straightness. 

It is for this reason that a new set of metamodels based in the Box–Behnken design have also been developed considering the possible variations of the blind holes’ tolerances. This type of experimental design is appropriate when trying not to exceed the limits of safe operation, in order to avoid the rejection of a product due to a non-conformity [25]. Compliance with geometric tolerances guarantees the correct mechanical behavior of a product. Therefore, the design points for the analysis of the Von Mises stresses present in the crank arm cannot be outside the limits marked by the tolerance values.

**Figure 12:**
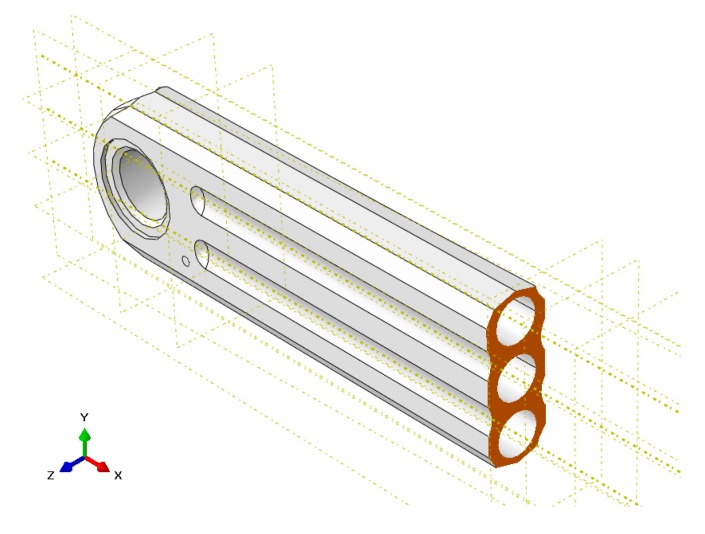
Numerical model of the crank arm sectioned in the *YZ* plane.

For the Box–Behnken design, the geometrical recommendations given by the international standard ISO 2768-1:1989 have been followed [26]. The tolerances considered in the sensitivity analysis are circularity, symmetry and straightness. Figure 12 shows a section view of the crank arm where the three central blind holes can be visualized. The drills have been created through cutting revolutions around the *X*-axis, therefore the straightness has been analyzed only in the *XY* plane. The tolerance of symmetry has been studied between the two blind holes located on both sides of the central one. On the other hand, the circularity has been evaluated by modifying the diameter of all the holes, in accordance with the tolerances recommended by the standard [26].

For the experimental design, it has been supposed that the confidence limits of the geometric tolerance are due to multiple measurements and represent the expanded uncertainty for a confidence level of 95%. Table 5 shows the experimental design space of the geometric tolerances. It is important to emphasize that in this phase of the study, it is only wanted to know the effect of the geometric tolerances in the design, therefore, the load has been considered constant and its value has been set at 918.63 N.

The probability density function for these sources of uncertainty has been considered as normal. As in the previous section, the uncertainty of the Von Mises stresses has been calculated from the random resampling of the possible values of the geometric tolerances in the constructed metamodel. The statistical magnitudes of the final stresses were calculated following the procedure recommended by GUM [14] for a confidence level of 95%.

**Figure 13:**
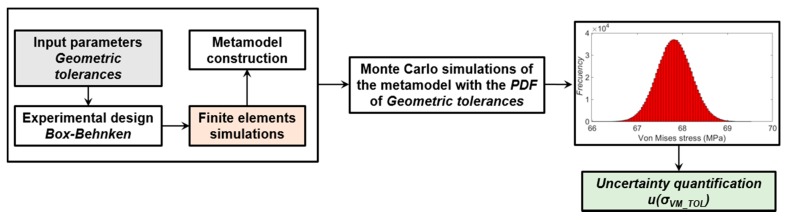
Uncertainty quantification procedure of the computational stress due to materials properties.

Figure 13 shows the procedure for the uncertainty quantification due to probable values of the geometric tolerances considered.

From the schematic representation shown in Figure 13, the Von Mises stress due to the uncertainty of the considered geometric tolerances and its associated typical uncertainty can been calculated as follows:

(12) $$\sigma_{VM\_TOL}=\frac{1}{M}\sum_{r=1}^{M}\sigma_{VM\_TOL,r},$$

(13) $$u\left(\sigma_{VM\_TOL}\right)=\sqrt{\frac{1}{M-1}\sum_{r=1}^{M}\left(\sigma_{VM\_TOL,r}-\sigma_{VM\_TOL}\right)},$$

where, *M* is the number of MMC trials and *σ_VM_TOL,r_* is the *r*th value of the numerical Von Mises stress from the metamodel simulation.

## 4. Model Validation Procedure

The model validation is the process of determining the level of compliance of the model with the real specimen for the required operation conditions. The goal of the model validation is to ensure that the mathematical or computational model is successfully defined by quantifying the quality of the predictive response of the model by comparison with experimental data [27]. Nowadays, the validation procedure demands the quantification of both experimental and computational model uncertainty. The models developed in the engineering field usually intent to reproduce the respond of the physical phenomenon according to the accuracy required. Therefore, the validation methodology can be developed with a predefined acceptable error due to simplification of the computational model or some boundary conditions, which is not taken into account [13].

The validation technique used in the present research follows the recommendations given by Hills [13]. According to this author, it can be concluded that the computational prediction of the Von Mises stress is consistent with the real test if the final experimental stress falls inside the range of values obtained from the random simulations of the computer model, for a confidence bound generally of 95%. To be able to verify this premise, it is necessary to quantify the uncertainty of both and then, as it is assumed that the measurement and the predictions are independent, it is possible to calculate a global combined uncertainty that will be equal to the square of the sum of the variance. Then, the global combined uncertainty is represented around the prediction results. 

The global uncertainty of the Von Mises stress for the strain gauge rosette was calculated with the following equation:

(14) $$u_{Global}=\sqrt{u^{2}(\sigma_{VM\_EXP})+u^{2}(\sigma_{VM\_MAT})+u^{2}(\sigma_{VM\_TOL})},$$

where, $u^{2}(\sigma_{VM\_EXP})$ is the final experimental uncertainty (9), $u^{2}(\sigma_{VM\_MAT})$ and $u^{2}(\sigma_{VM\_TOL})$ are the contributions of the computational model due to the material properties and geometric tolerances, Equations (11) and (13), respectively. On the other hand, the final computational Von Mises stress $\left(\sigma_{VM\_FEM}\right)$ will be the mean value of the stresses given by Equations (10) and (12).

## 5. Model Comparison

This section describes the procedure developed to verify the computational model, that is, to determine if it properly represents the behavior of the real crank arm under an experimental fatigue test.

To confirm that the crank arm model is able to predict the real test, two different comparisons have been carried between the test results and the numerical model. In the first one, five tests of 10 loading cycles were performed for two crank arm angle positions (±30°). In the second comparison it has been necessary to carry out a fatigue test of the real sample according to the standard [7]. The fatigue test was carried out under the same conditions as those used in the validation procedure, i.e., with the crank arm parallel to the horizontal plane. The test finished when the number of cycles required had been reached. 

The cycle fatigue test adopted by the standard [7] can be considered as low cycle fatigue test. This regime of alternating load should be between 10,000 and 100,000 cycles and it is characterized by high cyclic stress levels in excess of the endurance limit of the material [28]. 

In this research, the direct cyclic approach of Abaqus has been used to predict the fatigue life of the crank arm. This method allows to obtain the stabilized response of a structure subjected to periodic loading and it is an effective modelling technique suitable to perform low cycle fatigue predictions [29]. 

In bulk material, the cyclic loading simulates the progressive damage and failure due to the accumulation of plastic strains, which cause the initiation and subsequent propagation of cracks. The stabilized accumulated inelastic hysteresis strain energy per cycle characterizes the damage initiation and evolution in the simulated material [29]. 

In order to perform the fatigue analysis, it has been necessary to specify the inelastic behavior of the aluminum 7075-T6 used for the crank arm and its linear elastic properties. The fatigue analysis allows to verify if the stress is high enough to produce inelastic deformation and if this can cause the material failure before completing 100,000 load cycles. Table 6 shows typical values of the Yield stress and the plastic strain of the aluminum 7075-T6.

For the simulation of the fracture of the crank arm, a ductile damage initiation criterion has been used. This algorithm is suitable for predicting the onset of damage of metals due to nucleation, growth, and coalescence of voids [30]. The model criterion assumes that the equivalent plastic strain at the onset of damage is a function of the stress triaxiality *ƞ* and strain rate [29]. In this research, a stress triaxiality *ƞ* = 0.33 was assumed according to the literature review [31]. The fracture strain introduced was 0.11 and was selected according to [18] from the same source of typical properties shown in Table 6. 

Once the damage initiation criterion has been reached, due to the progressive degradation of the material stiffness, the next step is to specify the damage material evolution. This was assumed as a linear evolution of the fracture with the equivalent plastic displacement (Equation (15)).

(15) $${\dot{\bar{u}}}^{pl}=L{\dot{\bar{\varepsilon }}}^{pl},$$

where, *L* is the characteristic length of the element and ${\dot{\bar{\varepsilon }}}^{pl}$ is the equivalent plastic strain [29]. 

## 6. Results and Discussion

### 6.1. Experimental Results

**Figure 14:**
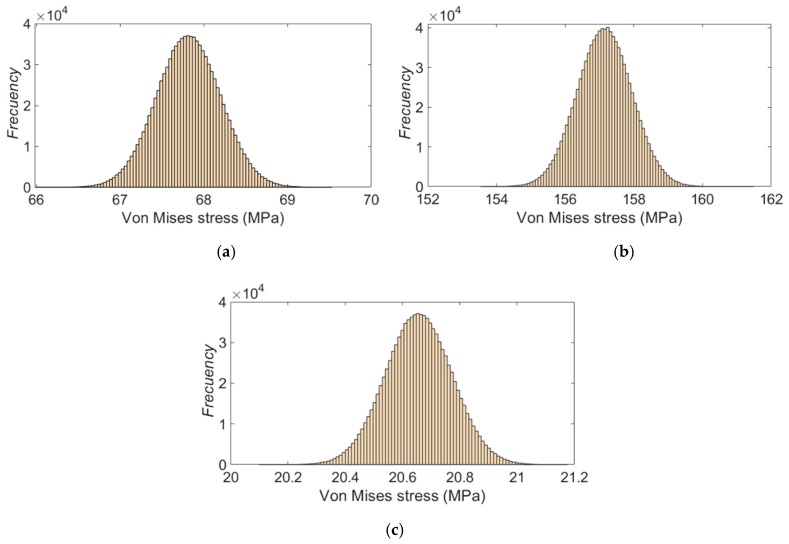
Experimental stress distribution for the strain gauge rosette: (**a**) #1, (**b**) #2 and (**c**) #3.

Figure 14 shows the histogram distribution of the possible values of the Von Mises stress for each strain gauge rosette, calculated according to the procedure explained in Section 2.3.1. The histograms were constructed using the final standard uncertainty (9). As can be seen, the behavior of the stress’ distribution corresponds to a normal probability density function. 

Table 7 shows the final values of the experimental Von Mises stresses, the combined uncertainty due the measurement technique and the experimental standard uncertainty of the strain gauge rosettes bonded on the crank arm surface. According to the magnitude of the relative uncertainty of the Von Mises stress *u_rel_*, it can be inferred that the test conditions have remained virtually unchanged in all the trials, so it is possible to ensure the precision of the experimental results. It was also verified that the uncertainty due to the measurement technique (7) represents approximately 50% to 60% of the value of $u(\sigma_{VM\_EXP})$.

### 6.2. Sensitivity of the FEM Model to the Uncertainty of the Material Properties and Load

Table 8 shows the results of the simulations of the carried out factorial design, for each of the zones equivalent to the location of the strain gauges in the real model. 

The statistical Statgraphic Centurion software has been used to construct the metamodel from the simulation data. The statistical parameters resulting from the analysis of the variance ANOVA inform that the most significant variable in the areas of the strain gauge rosettes is the applied force. In the zones occupied by rosettes #1 and #3, the Poisson ratio also is a significant model parameter. 

**Figure 15:**
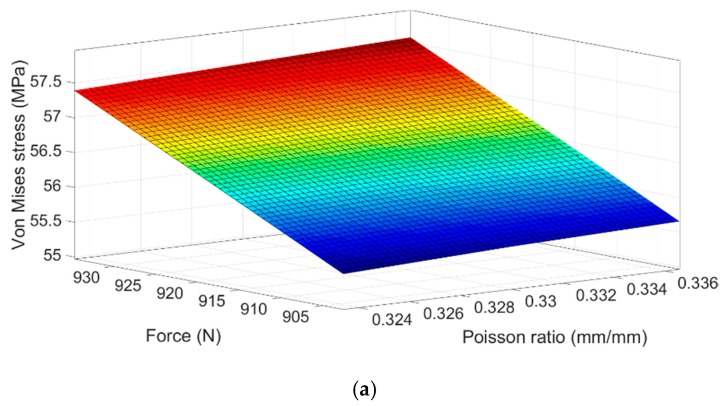

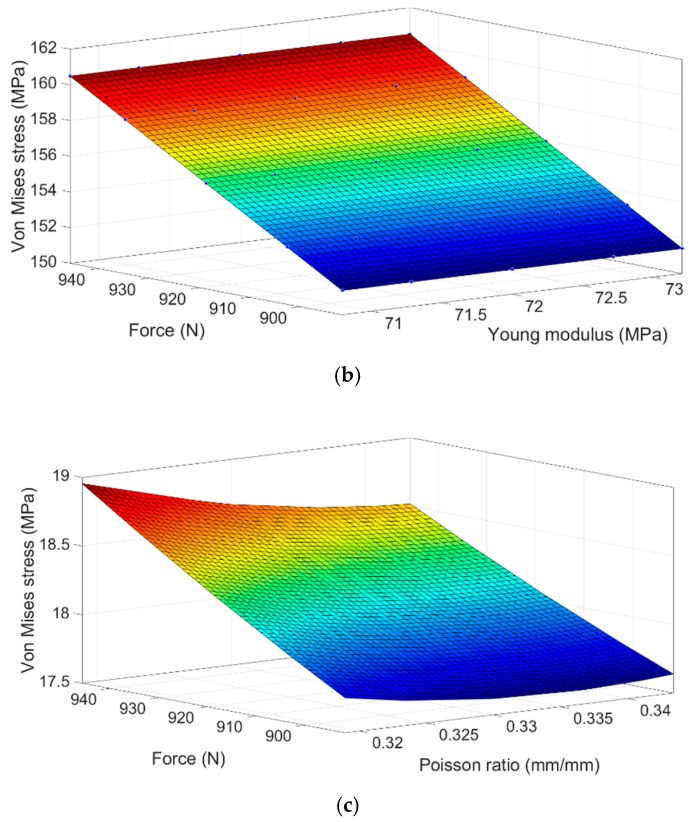
Response surface based in the material properties for the rosette zone: (**a**) #1, (**b**) #2 and (**c**) #3.

Figure 15 shows the response surfaces that allow to visualize the interaction effect of the variables that have the highest level of significance over the computational Von Mises stress in the three-zone analyzed. 

As can be seen in Figure 15, the analyzed zones are sensitive to variations in the value of the applied force. In rosette # 1 (Figure 15a), a higher applied force increases the stress by approximately 3.5%, whereas the increasing of the stress due to the Poisson ratio values does not exceed 0.3%. On the other hand, no interaction effect was observed in the zone of the rosette #2 (Figure 15b), so it can be concluded that this area is only sensitive to the uncertainty of the load. Figure 15c indicates that the combined effect of the load and the Poisson ratio affects the stress state in the zone occupied by rosette #3. A decrease of the Poisson ratio together with an increase of the applied force cause a higher Von Mises stress in this area (5%).

The metamodels obtained with the Statgraphic Centurion due to the material properties and the load can be expressed as follows:

(16) $$\sigma_{VM\_MATr1}=86.7079-0.0114457E-259.254\nu -0.0267184P+0.29105\nu P,$$

(17) $$\sigma_{VM\_MATr2}=0.213684+0.0136045E-0.571671\nu +0.168667P,$$

(18) $$\sigma_{VM\_MATr3}=78.1466-2.5608E-236.288\nu +0.139524P+7.75999E\nu -0.363488\nu P,$$

where $\sigma_{VM\_MATr1}$, $\sigma_{VM\_MATr2}$ and $\sigma_{VM\_MATr3}$ are the stresses in the zones occupied by the strain gauge rosettes #1, #2 and #3, respectively. The R-Squared statistics obtained are, in the same order, 99.8731%, 99.9997% and 98.0877%.

Table 9 presents the stress values and the uncertainty quantification calculated with the Equations (16)–(18) for the selected zones of the crank arm model, that have been estimated with the procedure explained in Section 3.1.1. It can be seen that the computational results are close to the experimental values shown in Table 7.

### 6.3. Sensitivity of the FEM Model to the Geometric Tolerances of the Blind Hole

Table 10 shows the simulation results for the sensitivity of the crank arm model due to the possible variation of the geometric tolerances of the blind hole.

An ANOVA analysis has been performed to study the variability of the stress values in the areas selected in the crank arm according to the variations of the geometric tolerances of the blind hole. In this case, the three factors taken into account have a probability value under 0.05, indicating that they are significant for a 95% confidence level. The straightness of the holes in the studied range of values is the factor with the smaller influence in the stress variation. 

**Figure 16:**
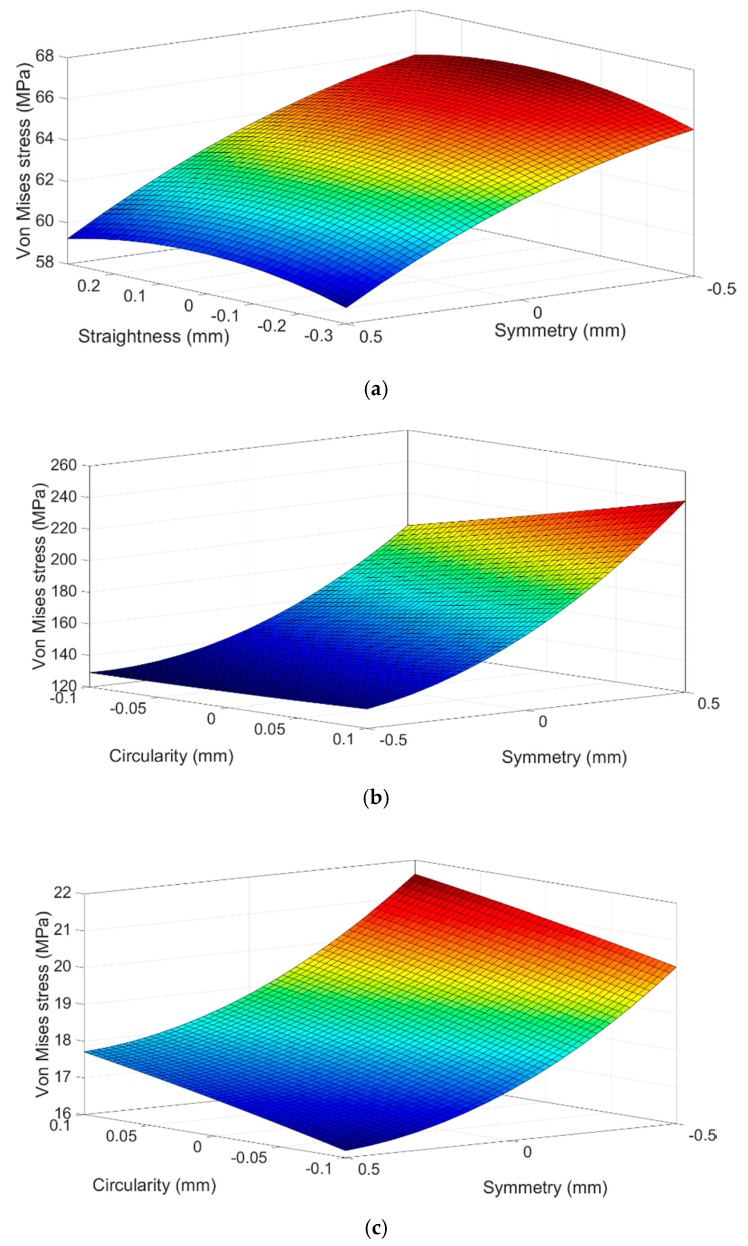
Response surface based on the geometric tolerance for the rosette zone: (**a**) #1, (**b**) #2 and (**c**) #3.

Figure 16 shows the response surfaces of the most important effects for the computational Von Mises stress, in the three-rosette area of the crank arm.

In Figure 16 it can be seen that the influence of tolerance of symmetry is present in the three response surfaces of the studied areas. In fact, this is the variable that has the greatest weight in the final value of the Von Mises stress. It has been observed that in both rosettes, #1 and #2, the interaction between the tolerances of circularity and symmetry causes a significant increase of the equivalent stress. The stress growth is slightly higher than 30% for rosette #1 and close to 45% for rosette #2. Therefore, it can be said that the uncertainties of the geometric tolerances produce a greater scattering of the stress compared to the properties of the material and applied load.

The metamodels obtained with the Statgraphic Centurion due to the geometrical tolerances can be expressed as follows:

(19) $$\begin{array}{ll} \sigma_{VM\_TOLr1} & =64.0214+0.981574S_{t}+1.75412C-6.45516S-10.2785S_{t}{}^{2}-57.7358C^{2}\\ & -3.43365S^{2}, \end{array}$$

(20) $$\sigma_{VM\_TOLr2}=155.142+7.37816S_{t}+112.789C+89.6232S+193.23CS+76.2555S^{2}$$

(21) $$\begin{array}{ll} \sigma_{VM\_TOLr3} & =18.1185-0.629863S_{t}+7.15902C-3.9873S+1.32681S_{t}{}^{2}-2.48884S_{t}S\\ & +3.40629S^{2} \end{array}$$

where $\sigma_{VM\_TOLr1}$, $\sigma_{VM\_TOLr2}$ and $\sigma_{VM\_TOLr3}$ are the stresses due to the geometric tolerance variation in the zone occupied by the strain gauge rosette #1, #2 and #3, respectively.

In this case, the R-Squared statistics obtained from the Statgraphic Centurion are, in the same order, explains 98.5214%, 99.1138% and 99.0538%.

Table 11 presents the final Von Mises stress values for the three rosettes, calculated with the metamodel expressions (19), (20) and (21) and their respective uncertainty, according to Section 3.1.2. It can be seen that Von Mises stress results are close to the previous computational results shown in Table 9 and to the experimental results of Table 7. However, the magnitude of the uncertainty due to the geometric tolerances is the highest. This confirms the importance of the compliance with the geometric tolerances during the manufacturing process of the real crank arm.

**Figure 17:**
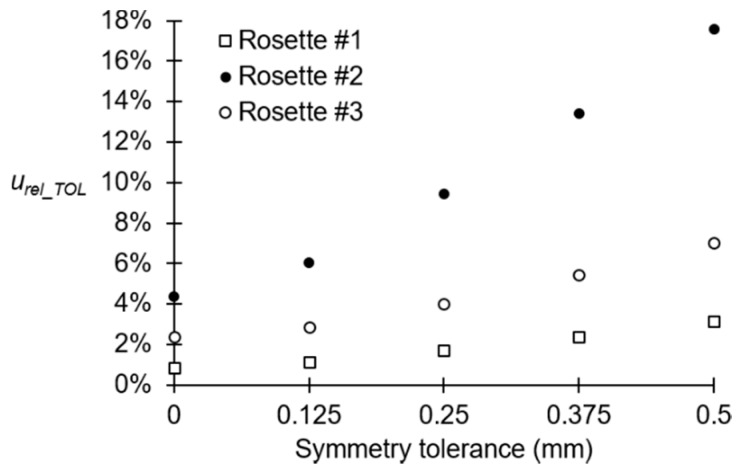
Evolution of the relative uncertainty of the Von Mises stress with respect to the symmetry tolerance.

In order to analyze the influence of the symmetry tolerance on the stresses of the rosettes zones, additional Monte Carlo simulations have been performed with the previous metamodels, for smaller symmetry tolerances. Figure 17 shows the evolution of the relative uncertainties of the numerical Von Mises stresses for the three rosettes. It can be seen that the area of rosette # 2 is the most sensitive to the increase of the symmetry tolerance and the rosette #1 is the less sensitive.

According to the results shown in Figure 17, geometric tolerances *S* ≤ 0.25 mm would be the most adequate, because the relative uncertainty is lower than 10%. This will minimize the stress variability of the produced crank arms, decreasing therefore the risk of premature failure. It can therefore be deduced that a precise manufacturing, together with a good metrological control are required to prevent premature crank arm failure.

The developed metamodels allows identifying the sources of uncertainty with the greatest influence, and to improve the crank arm model considering their impact. Additionally, it minimizes the computation time, since it is not necessary to perform multiple simple simulations of the finite element model to verify the effect of the possible variation of the input parameters into the model results.

### 6.4. Validation Results of the Finite Element Crank Arm

**Figure 18:**
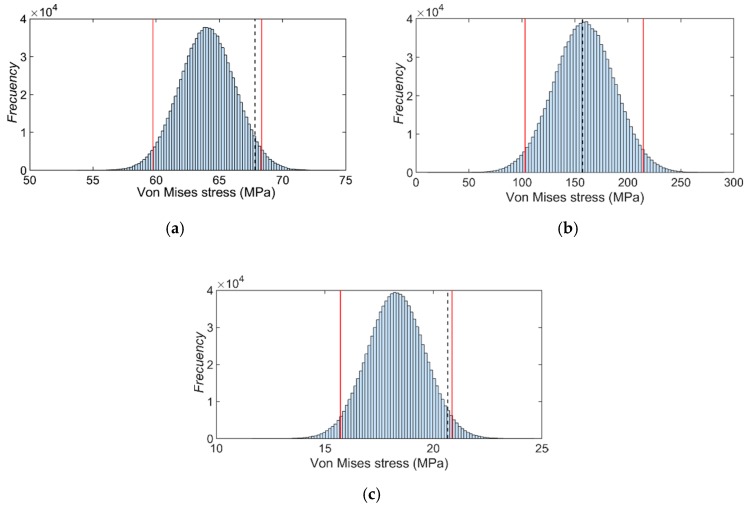
Results of the statistical validation of the crank arm model for the zone represented by rosette: (**a**) #1, (**b**) #2 and (**c**) #3.

Using the global uncertainty (14), that considers both the experimental and numerical uncertainties, it is possible to construct the histogram of the probable values of the model predictions using 10^6^ Monte Carlo simulations. Figure 18 shows the corresponding histograms for the three-rosette zone studied. The dashed line corresponds to the mean value of the experimental Von Mises stress. The 95% confidence limits of the global uncertainty are also included (red vertical lines). It can be seen that the experimental results are between the limits of the selected confidence level. This confirms that the numerical model in the areas analyzed, can reproduce the experimental case under study. 

Table 12 shows the mean values of the experimental and numerical stresses and the values of the global uncertainty of the Von Mises stress (14). Additionally, the relative error between the predicted values and the real ones have also been included. Although the global uncertainty in the area occupied by rosette #2 is high in comparison with the other areas, it has been observed that it is the one with the lowest relative error. Actually, the magnitude of the uncertainty by itself does not mean that the quality of the result is bad. What it indicates is that it is a potential failure zone and the sources of uncertainty must be controlled in order to minimize the risk of damage.

The statistical validation carried out in the present research work, allows to ensure that the numerical model is a good estimator of the real bicycle crank arm, under the test conditions performed. Additionally, it allows to identify the surface area most sensitive to boundary conditions (Rosette number 2) and the area where most attention must be paid, when the fatigue test is performed.

### 6.5. Confirmation of the Fatigue Behavior of the Crank Arm Model 

**Figure 19:**
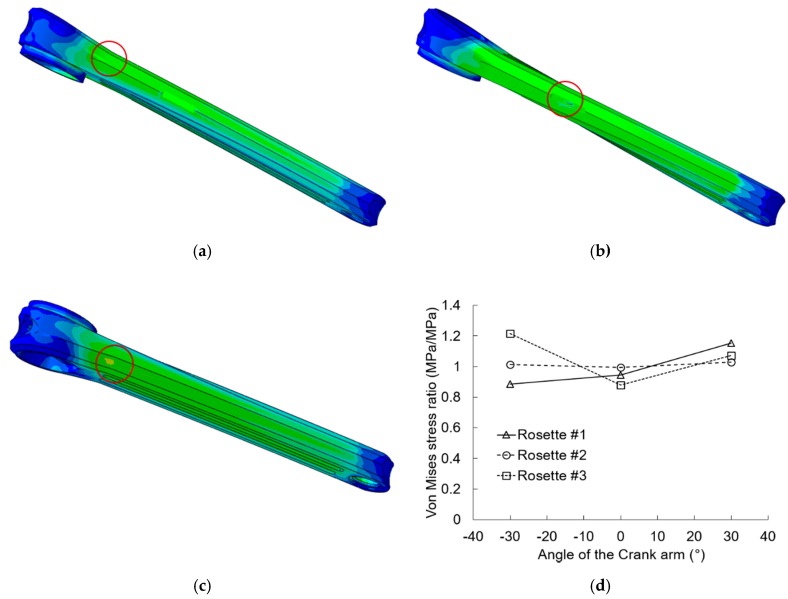
Verification of the stress behaviour of the crank arm for different angle position: (**a**) 30 degree, (**b**) 0 degree and (**c**) −30 degree and (**d**) ratio stress versus angle of the crank arm.

Figure 19 shows the results of the first comparison procedure. It can be seen that the location area of maximum Von Mises stress (red circle) changes with the angle of the crank arm. The potential damage zone is between the base of the crank arm and the area of rosette #2. The maximum stress can be on the same surface (Figure 19a) or on the opposite side (Figure 19c) from where rosette #2 is bonded. Moreover, in both cases, the maximum Von Mises stress is about 1.2 times higher than the stress of the rosette #2. Furthermore, it was confirmed that the greatest stress is found when the position angle is equal to zero, that is, when the crank arm axis is parallel to the horizontal plane.

Figure 19d shows the ratio between the computational stresses and the experimental ones for the three crank arm angles studied. It has been verified that, in all cases, the best estimation of the experimental stress corresponds to the area of rosette # 2. The prediction in this zone seems to be less sensitive to the angle of the crank arm, since the stress ratio remains almost constant. On the other hand, the greatest variability has been observed in the area occupied by rosette #3. However, the stress in this zone is low, so the probability of failure for the crank arm model in this zone is small.

Regarding the second developed comparison process (fatigue test), no structural failure of the real component has been detected; therefore, the crank arm complies with the minimum safety requirements given by the European Committee for Standardization [7]. Additionally, the instrumentation used allows to verifying the absence of permanent deformations during the fatigue test.

In the computational model, 110,000 cycles of load have been simulated with the direct cycle approach of Abaqus. The model used corresponds to the worst situation shown in Table 10, where the maximum stress of rosette #2 was observed (Run number 3). As for the real model, no plastic strain has been observed, therefore there are no signs of the beginning of structural failure. Once the statistical validation and structural verification procedures have been completed, it can be concluded that the finite element model of the crank arm correctly represents the real model, under the boundary conditions analyzed.

**Figure 20:**
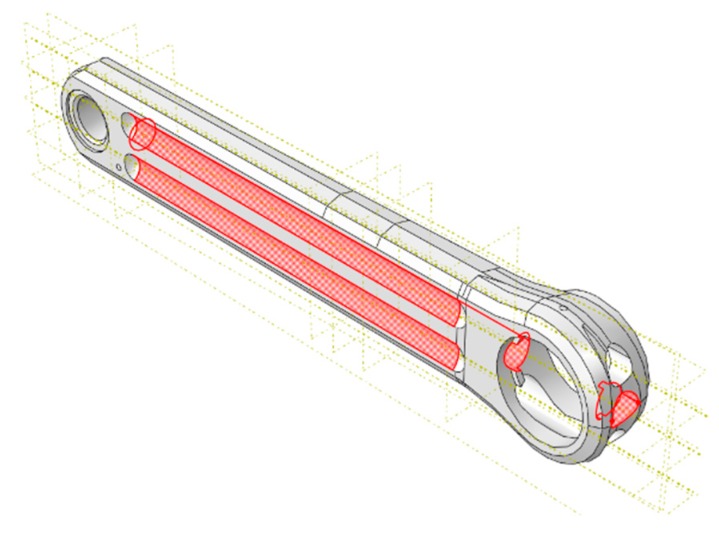
New crank arm optimized.

As each of the structural components of the bicycle must be as light as possible, but without affecting the strength to alternating loads, it has been decided to minimize the weight of the crank arm model. To reach this goal, a new crank arm with a larger diameter of the central hole and larger depth of the contoured grooves on both sides of the piece has been simulated for fatigue test (Figure 20). In this improved design, it has been considered that the crank arm meets the minimum requirements of the standard, but also that there are no permanent deformations in any area.

**Figure 21:**
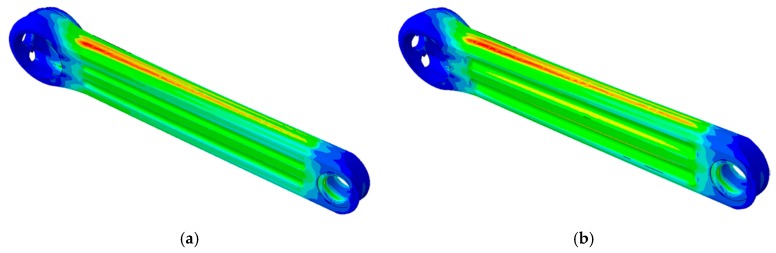
Comparison of the stress distribution map between: (**a**) the original model and (**b**) the new model.

Figure 21 shows the Von Mises stress distribution map of the original and the new crank arm. It has been observed that a decrease of 15% in the weight of the crank arm causes an increase of the maximum stress of 5.5%.

In Figure 21b it can be seen that the contoured grooves of the optimize crank are more stressed than the original model (Figure 21a). This area has the greatest increment of the final Von Mises stress (close to 26%) because of the reduction in thickness of the original model. Nevertheless, the critical area is still the one corresponding to zone of rosette #2. However, no inelastic behavior has been observed in the new design during the computational fatigue test performed. The maximum stress recorded in the new model was 471 MPa for an applied load of 1800 N. In this case, the safety factor value with respect to the yield stress (Table 1) is 1.07, whereas for the original design, the fatigue safety factor was 1.13.

**Figure 22:**
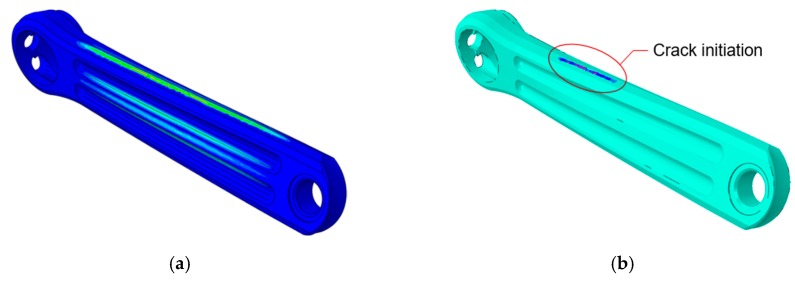
(**a**) Permanent deformation and (**b**) scalar stiffness degradation of the new crank arm model.

Additional simulations have been carried out on the improved model, in order to determine the magnitude of the load that causes its failure before completing the 100,000 cycles. Figure 22a shows the plastic strain distribution for a load 1.9 times higher than the standard requirements (3420 N), while Figure 22b shows the scalar stiffness degradation of the new model obtained for a load 2 times higher than the standard (3600 N). 

In the simulation results, it has been observed that a load of 3420 N produces permanent deformations in the crank arm in the area where rosette #2 was bonded and in the contoured grooves (Figure 22a). As the test recommended by the standard [7] only contemplates the fatigue failure performance, the component would meet the minimum safety requirements, because it would not fail before the 100,000 load cycles. Nevertheless, this can be a serious problem if it is not taken into account, because it can affect the structural behavior of the real crank and increase the probability of unexpected failure. This further justifies the inclusion, in the regulatory standard, [7] of the strain measurement in potential failure zones, as it has been demonstrated in this research work. On the other hand, it was observed that the structural degradation and the beginning of the failure of the crank arm occurs when the maximum cyclic load is 3600 N (Figure 22b). According to computational results, the crack initiation would begin at nearly 22,000 cycles. It can be inferred that the load causing the failure due to fatigue can probably be between the loads values mentioned above. 

## 7. Conclusions

In this paper, an aluminum crank arm modelled in Abaqus has been validated and verified with experimental results. The type of sensor used allows us to know the state of the deformations and therefore of the stresses with great precision due to the high repeatability shown in the tests. In addition, it facilitates the calculation of the uncertainties of the final measures since the expressions for the calculus of the potential sources of errors of this measurement technique are known. A complete uncertainty quantification has been carried out in order to compare the real model with the finite element model in the areas where damage usually occurs. It has been observed that the effect of the uncertainties of the elastic properties of the material on the stresses of the numerical model is negligible. In contrast, it was proved that the geometric tolerances of the central holes of the crank arm have the greatest effect, followed by the uncertainty of the testing machine. Therefore, an exhaustive metrological control of the analyzed tolerances and a precise manufacturing process is recommended in order to avoid premature failure of the component.

The result of the validation informs that the performed model can satisfactorily predict the behavior of the real crank arm in the studied zones. Additionally, the structural verification of the component made through the fatigue test, indicates that both the real and modelled crank arm meet the minimum safety requirements.

The validation and comparison procedure allows to develop a new model of the crank arm with less weight. In this case, it was possible to decrease the weight until 15% without the fatigue strength being seriously affected. The weight reduction can improve the performance of the bike at a professional level. Therefore, it can be confirmed that the quantification of the uncertainties allow a better knowledge of the analyzed component and a better control of the manufacturing process, since it helps to detect the areas that require special metrological control.

Based on the results obtained in this research work, it is recommended that the European Committee for Standardization consider, in the current standard, to check that permanent deformations do not appear in the potential failure zones of the bicycle component during the fatigue test. Since we have observed that, it is possible that a crank arm can fulfil with the minimum safety requirements of the standard but present areas with permanent deformations. This causes residual stresses that, in conjunction with the stochastics load conditions to which the crank arm is subjected in a real competition, may increase the risk of injury for the rider due to premature crank failure. For this, the classic resistive strain gages bonded in the areas where the failure usually occurs can be used, due to its high precision and easy installation.

## Figures and Tables

**Table 1:** Typical mechanical properties of the aluminum 7075-T6 [18].

Properties	Value	Unit
Tensile strength	572	MPa
Yield strength	503	MPa
Young Modulus	72	GPa
Poisson ratio	0.33	µm/µm

**Table 2:** Main error sources in strain gauges measurement technique [17].

Error Due to	Formula
Wheatstone bridge nonlinearity	$E_{w}={\hat{\varepsilon }}_{i}-\frac{2{\hat{\varepsilon }}_{i}}{2-F{\hat{\varepsilon }}_{i}},$ *ε_i_*: strain indicated by the strain data acquisition system. *F*: gauge factor.
Temperature	$E_{T}=\frac{2}{F\left[1+\alpha_{G}\left(T_{E}-T_{c}\right)\right]}\cdot \left[\varepsilon_{T/0}+\left(\alpha_{s(B)}-\alpha_{s(A)}\right)\cdot \left(T_{E}-T_{c}\right)\right],$ 2: gauge factor used by the manufacturer to estimate the thermal output of the strain gauge. *α_G_*: temperature coefficient of the gauge factor. *ε_T/0_*: thermal output or apparent strain. *α_s(B)_*: coefficient of thermal expansion of the test material. *α_s(A)_*: coefficient of thermal expansion of the material used by the strain gauge manufacturer. *T_E_*, *T_C_*: Temperatures of the test piece and the room-temperature on the gauge package data label, respectively.
Transverse sensitivity	$E_{TS,1}={\hat{\varepsilon }}_{1}-\left\{\frac{1-\nu_{0}K_{t}}{1-K_{t}{}^{2}}\cdot \left[{\hat{\varepsilon }}_{1}-K_{t}{\hat{\varepsilon }}_{2}\right]\right\},$ $E_{TS,2}={\hat{\varepsilon }}_{2}-\left\{\frac{1-\nu_{0}K_{t}}{1-K_{t}{}^{2}}\cdot \left[{\hat{\varepsilon }}_{2}-K_{t}{\hat{\varepsilon }}_{1}\right]\right\},$ $E_{TS,3}={\hat{\varepsilon }}_{3}-\left\{\frac{1-\nu_{0}K_{t}}{1-K_{t}{}^{2}}\cdot \left[{\hat{\varepsilon }}_{3}-K_{t}\left({\hat{\varepsilon }}_{1}+{\hat{\varepsilon }}_{2}-{\hat{\varepsilon }}_{3}\right)\right]\right\},$ ${\hat{\varepsilon }}_{1}$,${\hat{\varepsilon }}_{2}$,${\hat{\varepsilon }}_{3}$: uncorrected strain of the gauges 1, 2 and 3 of the stacked grid 3-element strain gauge rosettes. The axis of grid 2 be 90° away from that of grid 1; and grid 3 be 45° away, in the same rotational direction (counterclockwise). *K_t_*: transverse sensitivity coefficient. *ν*_0_: 0.285

**Table 3:** Values, uncertainty and probability density functions of all the variables.

Variable	Value	Units	Tolerance	Uncertainty *u*	PDF
${\hat{\varepsilon }}_{i}$	Varies	µε	±5.00 × 10^−1^	2.89 × 10^−1^	Gaussian
*F*	2.14	Dimensionless	±1.00 × 10^−2^	5.77 × 10^−3^	Rectangular
*K_t_*	2.00 × 10^−3^	Dimensionless	±2.00 × 10^−3^	5.77 × 10^−4^	Rectangular
*v*_0_	0.285	Dimensionless	±1.00 × 10^−2^	5.77 × 10^−3^	Rectangular
*T_E_*	23	°C	±1.00	5.77 × 10^−1^	Rectangular
*ε_T_*	0	µm/m	−	8.50 × 10^−1^ (µm/m)/°C	Gaussian
*P*	Varies	kN	−	8.80 × 10^−3^*P*	Gaussian
*T_C_*	23	°C	−	−	
*E*	72	GPa		3.6 × 10^−1^	Gaussian
*v*	0.33	µm/µm		3.3 × 10^−3^	Gaussian

**Table 4:** Experimental design based on the materials properties and applied load.

Run Number	*E* (GPa)	*v* (µm/µm)	*P* (N)
1	71.28	0.3234	902.55
2	72.72	0.3434	902.55
3	71.28	0.3366	902.55
4	72.72	0.3366	902.55
5	71.28	0.3234	934.71
6	72.72	0.3234	934.71
7	71.28	0.3366	934.71
8	72.72	0.3366	934.71
9	70.7891	0.33	918.63
10	73.2109	0.33	918.63
11	72	0.3189	918.63
12	72	0.3411	918.63
13	72	0.33	891.587
14	72	0.33	918.63
15	72	0.33	945.673
16	72	0.33	918.63

**Table 5:** Experimental design based in the geometric tolerance values.

Run Number	Circularity C (mm)	Symmetry S (m/m)	Straightness St (mm)
1	0.0	0.0	0.0
2	0.0	−0.5	0.3
3	0.1	0.5	0.0
4	0.0	0.0	0.0
5	−0.1	0.0	0.3
6	0.0	−0.5	−0.3
7	0.1	0.0	−0.3
8	0.0	0.0	0.0
9	0.1	−0.5	0.0
10	−0.1	0.5	0.0
11	0.0	0.5	−0.3
12	−0.1	−0.5	0.0
13	−0.1	0.0	−0.3
15	0.1	0.0	0.3
16	0.0	0.5	−0.3

**Table 6:** Typical plastic properties of the aluminum 7075-T6 [18].

Yield Stress (MPa)	Plastic Strain
513	0
524	0.002875
543	0.012875
554	0.022875
563	0.032875
569	0.042875
575	0.052875
578	0.068750
582	0.072875

**Table 7:** Final experimental stress calculated from the strain gauge measure.

Rosette Number	$\sigma_{VM\_EXP}\;(\mathrm{MPa})$	$u(\sigma_{VM\_EXP})\;(\mathrm{MPa})$	$u_{rel}=100\frac{u(\sigma_{VM\_EXP})}{\sigma_{VM\_EXP}}\;(\%)$
#1	67.82	0.74	1.09
#2	157.15	1.42	0.91
#3	20.66	0.18	0.87

**Table 8:** Summary of the simulations results based in Central Composite Design (CCD) of the materials properties and load.

Run Number	*E* (GPa)	*v* (µm/µm)	*P* (N)	$\sigma_{VM\_MAT}\;(\mathrm{MPa})$		
				Rosette #1	Rosette #2	Rosette #3
1	71.28	0.3234	902.55	62.84	153.23	17.93
2	72.72	0.3434	902.55	62.84	153.25	17.93
3	71.28	0.3366	902.55	62.98	153.22	17.74
4	72.72	0.3366	902.55	62.98	153.23	17.74
5	71.28	0.3234	934.71	65.06	158.65	18.56
6	72.72	0.3234	934.71	65.06	158.67	18.57
7	71.28	0.3366	934.71	65.21	158.64	18.37
8	72.72	0.3366	934.71	65.20	158.66	18.37
9	70.7891	0.33	918.63	64.03	155.94	18.15
10	73.2109	0.33	918.63	64.02	155.97	18.15
11	72	0.3189	918.63	63.90	155.95	18.32
12	72	0.3411	918.63	64.14	155.94	17.99
13	72	0.33	891.587	62.15	151.39	17.62
14	72	0.33	918.63	64.02	155.95	18.15
15	72	0.33	945.673	65.89	160.51	18.68
16	72	0.33	918.63	64.02	155.95	18.15

**Table 9:** Uncertainty quantification of the crank arm model due to the material properties and the applied load.

Rosette Number	$\sigma_{VM\_MAT}\;(\mathrm{MPa})$	$u(\sigma_{VM\_MAT})\;(\mathrm{MPa})$	$u_{rel}=100\frac{u(\sigma_{VM\_MAT})}{\sigma_{VM\_MAT}}\;(\%)$
#1	64.04	0.56	0.87
#2	155.95	1.36	0.87
#3	18.13	0.16	0.90

**Table 10:** Summary of the simulations results based in the Box–Behnken design of the geometric tolerances.

Run Number	Circularity C (mm)	Symmetry S (m/m)	Straightness St (mm)	$\sigma_{VM\_TOL}\;(\mathrm{MPa})$		
				Rosette #1	Rosette #2	Rosette #3
1	0.0	0.0	0.0	64.02	155.95	18.15
2	0.0	−0.5	0.3	66.20	129.91	21.44
3	0.1	0.5	0.0	60.18	246.27	17.77
4	0.0	0.0	0.0	64.02	155.95	18.15
5	−0.1	0.0	0.3	62.70	147.85	17.24
6	0.0	−0.5	−0.3	65.41	128.48	20.70
7	0.1	0.0	−0.3	62.12	156.95	19.24
8	0.0	0.0	0.0	64.02	155.95	18.15
9	0.1	−0.5	0.0	65.75	135.00	21.75
10	−0.1	0.5	0.0	59.22	196.82	16.06
11	0.0	0.5	−0.3	58.42	214.24	17.54
12	−0.1	−0.5	0.0	65.20	124.19	20.21
13	−0.1	0.0	−0.3	62.38	146.27	17.94
14	0.1	0.0	0.3	62.87	167.11	18.44
15	0.0	0.5	−0.3	58.9	218.75	16.78

**Table 11:** Uncertainty quantification of the crank arm model due to the geometric tolerance of the blind hole.

Rosette Number	$\sigma_{VM\_TOL}\;(\mathrm{MPa})$	$u(\sigma_{VM\_TOL})\;(\mathrm{MPa})$	$u_{rel\_TOL}=100\frac{u(\sigma_{VM\_TOL})}{\sigma_{VM\_TOL}}\;(\%)$
#1	63.24	1.99	3.1
#2	161.81	28.40	17.6
#3	18.45	1.29	7.0

**Table 12:** Final experimental and numerical Von Mises stress for the three studied zones.

Rosette Number	$\sigma_{VM\_FEM}\;(\mathrm{MPa})$	$\sigma_{VM\_EXP}$ (MPa)	$u_{Global}$ (MPa)	$\%Error=100\frac{\sigma_{VM\_FEM}-\sigma_{VM\_EXP}}{\sigma_{VM\_EXP}}$
#1	63.62	67.82	2.19	−6.19
#2	158.85	157.15	28.47	1.10
#3	18.29	20.66	1.31	−11.47

## References

[1] Oosterhuis H. (2016). Cycling, modernity and national culture. Soc. Hist..

[2] Vairakanna P.L., Chandran M. (2017). Design and material study of race bike crank. Int. J. Eng. Sci. Res. Technol..

[3] Rontescu C., Cicic T.D., Amza C.G., Chivu O. (2015). Choosing the optimum material for making a bicycle frame. Metalurgija.

[4] Davis R., Hull M.L. (1981). Design of aluminum bicycle frames. J. Mech. Des..

[5] Calvo José A., Álvarez-Caldas C., Santos S., Gutiérrez R. (2018). Influence of anodized depth on fatigue life for bicycle cranks. Eng. Fail. Anal..

[6] McKenna S.P., Hill M.R., Hull M.L. (2003). Methods for fatigue testing off-road bicycle handlebars based on assembly effects using two different stem designs. J. Test. Eval..

[7] European Commitee for Standardization (2005). Racing Bicycles—Safety Requirements and Test Methods.

[8] Lin C.C., Huang S.J., Liu C.C. (2017). Structural analysis and optimization of bicycle frame designs. Adv. Mech. Eng..

[9] Madivalar1a C.N., Shay1b T., Kolekar S. (2018). Fatigue failure analysis of bike crank arm using solid works. J. Mech. Eng. Res. Dev..

[10] Ramos J.A.C., lvarez-Caldas C., Quesada A., Román J.L.S. (2016). Determining the stress distribution in a bicycle crank under in-service loads. Exp. Tech..

[11] Bini R.R., Hume P.A., Cerviri A. (2011). A comparison of cycling SRM crank and strain gauge instrumented pedal measures of peak torque, crank angle at peak torque and power output. Proced. Eng..

[12] Balbinot A., Milani C., da Nascimento J.S.B. (2014). A new crank arm-based load cell for the 3D analysis of the force applied by a cyclist. Sensors.

[13] Hills R., Trucano T. (1999). Statistical Validation of Engineering and Scientific Models: Background.

[14] Chen W., Baghdasaryan L., Buranathiti T., Cao J. (2004). Model validation via uncertainty propagation. AIAA J..

[15] Doebling S.W., Hemez F.M., Schultze J.F., Cundy A.L. A metamodel-based approach to model validation for nonlinear finite element simulations. Proceedings of the A Conference and Exhibition on Structural Dynamics.

[16] Hariri-Ardebili M.A., Seyed-Kolbadi S.M., Noori M. (2018). Response surface method for material uncertainty quantification of infrastructures. Shock Vib..

[17] Watson R.B., Sharpe W.N. (2008). Bonded Electrical Resistance Strain Gauges.

[18] Muraca R.F., Whittick J.S. (1972). Materials Data Handbook.

[19] Gabauer W. (2000). The Determination of Uncertainties in Tensile Testing. Manual of Codes of Practice for the Determination of Uncertainties in Mechanical Tests on Metallic Materials.

[20] Montero W., Farag R., Díaz V., Ramirez M., Boada B.L. (2011). Uncertainties associated with strain-measuring systems using resistance strain gauges. J. Strain Anal. Eng. Des..

[21] Joint Committee for Guides in Metrology (2008). Evaluation of Measurement Data—Guide to the Expression of Uncertainty in Measurement.

[22] Joint Committee for Guides in Metrology (2008). JCGM 101: Evaluation of Measurement Data—Supplement 1 to the “Guide to the Expression of Uncertainty in Measurement”—Propagation of Distributions Using a Monte Carlo Method.

[23] Nishio M., Marin J., Fujino Y. (2012). Uncertainty quantification of the finite element model of existing bridges for dynamic analysis. J. Civ. Struct. Heal. Monit..

[24] Mangado N., Piella G., Noailly J., Pons-Prats J., Ballester M.Á.G. (2016). Analysis of uncertainty and variability in finite element computational models for biomedical engineering: Characterization and propagation. Front. Bioeng. Biotechnol..

[25] Myers R.H., Montgomery D.C., Anderson-Cook C.M. (2011). Response Surface Methodology: Process and Product Optimization Using Designed Experiments.

[26] International Organization for Standardization (1989). General Tolerances—Part 1: Tolerances for Linear and Angular Dimensions without Individual Tolerance Indications.

[27] Ben T.H., Scott Doebling W., Francois Hemez M., Mark Anderson C., Jason Pepin E., Edward Rodriguez A. (2004). Concepts of Model Verification and Validation.

[28] McLean M. (2014). Encyclopedia of materials science and technology. Int. Met. Rev..

[29] Simulia D.S. (2016). Abaqus Documentation.

[30] Hooputra H., Gese H., Dell H., Werner H. (2004). A comprehensive failure model for crashworthiness simulation of aluminium extrusions. Int. J. Crashworth..

[31] Kut S. (2009). State of stress identification in numerical modeling of 3D issues. Arch. Metall. Mater..

